# Metabolomic profiling to characterize acute intestinal ischemia/reperfusion injury

**DOI:** 10.1371/journal.pone.0179326

**Published:** 2017-06-29

**Authors:** Rachel G. Khadaroo, Thomas A. Churchill, Victor Tso, Karen L. Madsen, Chris Lukowski, Saad Y. Salim

**Affiliations:** 1 Department of Surgery, Faculty of Medicine and Dentistry, University of Alberta, Edmonton, Alberta, Canada; 2 Division of General Surgery, Faculty of Medicine and Dentistry, University of Alberta, Edmonton, Alberta, Canada; 3 Division of Critical Care Medicine, Faculty of Medicine and Dentistry, University of Alberta, Edmonton, Alberta, Canada; 4 Centre of Excellence for Gastrointestinal Inflammation & Immunity Research (CEGIIR), Faculty of Medicine and Dentistry, University of Alberta, Edmonton, Alberta, Canada; Instituto de Investigacion Sanitaria INCLIVA, SPAIN

## Abstract

Sepsis and septic shock are the leading causes of death in critically ill patients. Acute intestinal ischemia/reperfusion (AII/R) is an adaptive response to shock. The high mortality rate from AII/R is due to the severity of the disease and, more importantly, the failure of timely diagnosis. The objective of this investigation is to use nuclear magnetic resonance (NMR) analysis to characterize urine metabolomic profile of AII/R injury in a mouse model. Animals were exposed to sham, early (30 min) or late (60 min) acute intestinal ischemia by complete occlusion of the superior mesenteric artery, followed by 2 hrs of reperfusion. Urine was collected and analyzed by NMR spectroscopy. Urinary metabolite concentrations demonstrated that different profiles could be delineated based on the duration of the intestinal ischemia. Metabolites such as allantoin, creatinine, proline, and methylamine could be predictive of AII/R injury. Lactate, currently used for clinical diagnosis, was found not to significantly contribute to the classification model for either early or late ischemia. This study demonstrates that patterns of changes in urinary metabolites are effective at distinguishing AII/R progression in an animal model. This is a proof-of-concept study to further support examination of metabolites in the clinical diagnosis of intestinal ischemia reperfusion injury in patients. The discovery of a fingerprint metabolite profile of AII/R will be a major advancement in the diagnosis, treatment, and prevention of systemic injury in critically ill patients.

## Introduction

Sepsis and septic shock are the leading causes of death in critically ill patients [1]. The gastrointestinal tract serves as an important defence barrier for the body. In critically ill patients, the gastrointestinal tract can become a major pathogenic source of bacteria and inflammatory mediators that can lead to a septic shock [2–5]. Acute intestinal ischemia/reperfusion (AII/R) is an adaptive response to shock. Mortality from shock due to AII/R are 60–90% and have not changed since the 1940s [6–8]. The high mortality rate for AII/R is due to the severity of the disease and, more importantly, the failure of a timely diagnosis. Rapid diagnosis of intestinal ischemia would allow for earlier intervention which would result in: 1) avoidance of surgery by earlier aggressive fluid resuscitation, 2) if surgery was needed, it would occur earlier, resulting in lower postoperative morbidity and mortality.

**Fig 1:**
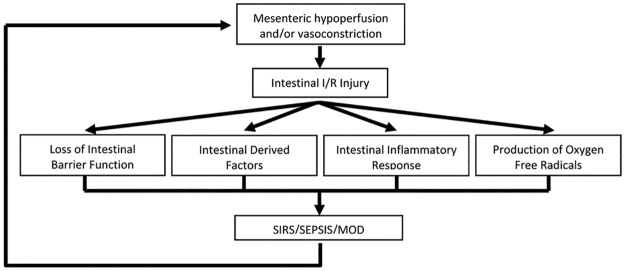
Cycle of mesenteric hypoperfusion resulting in sepsis, septic shock and MOD. Following systemic shock there is a decrease intestinal mesenteric perfusion resulting in gastrointestinal injury. This can result in loss of intestinal barrier function, release of intestinal derived factors, production and release of cytokines and free radicals. The culmination of these events can lead to sepsis, septic shock and multiple organ dysfunction (MOD) which contributes to a vicious cycle of local and distant organ injury.

Patients may experience shock as a consequence of the body’s adaptive response to injury. This results in the vasoconstriction and hypoperfusion of mesenteric arteries. This hypoxic condition damages the intestines, which is further exacerbated by subsequent reperfusion leading to AII/R. AII/R induced intestinal mucosal barrier damage and the following inflammation results in the translocation of micro-organisms and endotoxins from the gut lumen into the systemic circulation [9–12], in combination with the production of oxygen-derived free radicals [13, 14], proinflammatory cytokines [15], and other undefined intestinally derived factors [16, 17]. In a murine model of AII/R, we demonstrated local and systemic cytokine increase [18]. The triggered signaling cascade has the potential to escalate into a vicious cycle of continuously increasing intestinal permeability (Fig 1), resulting in local small bowel and distant organ lung injuries [18]. All of these factors ultimately contribute to the onset of sepsis, septic shock and multiple organ dysfunction (MOD).

Several treatment modalities have shown the importance of intestinal mucosal integrity in preventing infection. A meta-analysis comparing enteral with parenteral nutrition found a significant reduction in infectious complications and a trend towards decreased mortality [19]. Glutamine, a central amino acid required for intestinal integrity, improves the prognosis in critically ill burn and trauma patients, presumably by maintaining the intestinal barrier and reducing the frequency of infections [19, 20]. It is possible to decrease the bacterial load of the gut through selective digestive tract decontamination consisting of intravenous and topical application of antibiotics in the oropharynx and stomach. In critically ill septic patients, selective digestive tract decontamination resulted in a significant reduction of 28-day mortality [21]. Additionally, we have shown that pretreatment with probiotics in an animal model reduced small bowel tissue injury in AII/R [18]. These studies provide corroborating evidence of the involvement of intestinal microflora in the pathogenesis of sepsis. The knowledge that the microbiota plays an essential role in systemic inflammation caused by AII/R supports the utility of creating a metabolomic profile.

While genomics and proteomics have become useful tools to examine gene and protein expression, metabolomics provides data on an organism’s cellular metabolism (i.e. production of amino acids, peptides, lipids, carbohydrates). The metabolite profile of an organism can vary as a result of environment or disease state. In addition, metabolomics can be used to examine alterations in the intestinal microbiota which may result from infection or metabolic syndromes [22–24]. Studies have proposed the use of metabolomics to diagnosis myocardial infarction [25] and kidney ischemia/reperfusion (I/R) injury following transplantation [26, 27]. One animal study examined late intestinal ischemia without a reperfusion phase [28]. However to date, there have been no studies applying metabolomics profiling to study AII/R.

The purpose of this investigation was to find correlations between the urinary metabolite profile and the development of AII/R injury which will aid in the diagnosis and understanding the metabolite fingerprint associated with early and late AII/R injury. This animal investigation will provide proof-of-concept that can be applied to future studies examining the role of metabolomics in the patient population.

## Materials and methods

### Animal model

This study was reviewed and approved by the University of Alberta Animal Care and Use Committee and carried out in accordance with guidelines from the Canadian Council on Animal Care (Protocol number: 643/06/11/D). Adult 129/SvEv mice were anesthetised with an intraperitoneal injection of ketamine (120 mg/kg) and xylazine (16 mg/kg) and a laparotomy was performed to expose the superior mesenteric artery. Internal body temperature was maintained at 37°C with overhead heat lamps and/or a heating pad. Animals were subjected to intestinal ischemia as described before [29]. Briefly, occlusion of the superior mesenteric artery was applied with a vascular microclamp (10g pressure). Time points representative of early (30 min, n = 4) and late (60 min, n = 4) intestinal ischemic injury were performed followed by reperfusion via removal of microclamp for 2 hrs. Sham animals were also subjected to laparotomy for 3 hrs however, no microclamp was applied to the mesenteric artery (0 min ischemia, n = 3). Mice were euthanized at the end of the 2 hrs of reperfusion. Organs were harvested for assessment and urine samples were collected directly from the bladder using a 26½G needle. Collected urine were supplemented with 1/10 vol of 0.5% (w/v) sodium azide (NaN_3_) to prevent bacterial contamination. Samples were given a unique identifiers and then frozen at -80°C until further analysis. The NMR operator (VT) was blinded to the samples prior to processing and analysis.

### Myeloperoxidase (MPO) assay

Intestinal tissues were rinsed in PBS and homogenized in 800 *μ*L ice-cold potassium phosphate buffer (50 mM KH_2_PO_4_ [pH 6.0] containing 50 mM hexadecyltrimethylammonium bromide (HTAB)) with a hand-held homogenizer for 15 seconds. Samples were sonicated for 15 seconds, frozen in liquid nitrogen and thawed. Following centrifugation (21,000*g*, 20 mins, 4°C), the supernatants were collected and the pellets were sonicated again in 400 *μ*L of potassium phosphate buffer (pH 6.0) containing 50 mM HTAB, frozen in liquid nitrogen, thawed and centrifuged. Centrifugation, sonication, freezing and thawing was repeated an additional time (3 in total) and all the supernatents were pooled together. The pooled supernatents were frozen at -80°C until MPO activity was analyzed. The protein content of the samples was determined using the Pierce Coomassie BSA Protein Assay (Pierce, Rockford, IL). MPO activity was assessed by addition of 10 *μ*L sample to 290 *μ*L assay buffer (50 mM KH_2_PO_4_, pH 6.0 with 0.167 mg/mL o-dianisidine dihydrochloride and 0.0005% H_2_O_2_). The reaction kinetics (V_max_) were analyzed every minute over 30 minutes at 460 nM on a microplate reader. The V_max_ value was divided by the protein concentration in order to compare MPO activity across different samples.

### Nuclear magnetic resonance (NMR) analysis

Urine samples were centrifuged (4.7k x g) through rehydrated Nanosep Omega 3Kspin filters (Pall Corporation, Port Washington, NY) to remove debris/protein. Collected urine were diluted to 635 *μ*L and 65 *μ*L of internal standard (5 mM sodium 2, 2-dimethyl-2-silapentane-5-sulfonate-*d*6; DSS-*d*6) plus 0.2% sodium azide in 100% D_2_O (Chenomx internal standard, Chenomx Inc., Edmonton, Canada). The pH of each urine sample was adjusted to 6.8 ± 0.1 with HCl or NaOH. A 700 *μ*L aliquot was placed in a 5 mm NMR tube (Wilmad, Buena, NJ) and NMR analysis was performed on the same day. One-dimensional NMR spectra of urine samples were acquired using the first increment of the standard ‘Nuclear OverHauser Enhancement Spectroscopy’ (NOESY) pulse sequence on a four-channel Varian INOVA 600 MHz NMR spectrometer equipped with a triax-gradient 5 mm HCN probe as previously described [30, 31]. Quantification of metabolites was achieved using Chenomx NMR Suite 7.0 (Chenomx Inc.). The Chenomx compound library contains 297 compounds. The NMR variables (metabolites) derived from spectral analysis were log_10_ transformed, mean centered, and unit variance scaling was applied. Principal component analysis (PCA) and orthogonal partial least squares-discriminant analysis (OPLS-DA) was conducted using SIMCA P11.0 (Umetrics, Umeå, Sweden). Individual metabolites (sham *vs*. AII/R) were analyzed by Mann-Whitney U test over the time course of ischemia. The univariate test was conducted using the Prism 4.0c (GraphPad Software Inc., San Diego, CA).

## Results

### Intestinal myeloperoxidase (MPO) as an index of inflammation/injury

**Fig 2:**
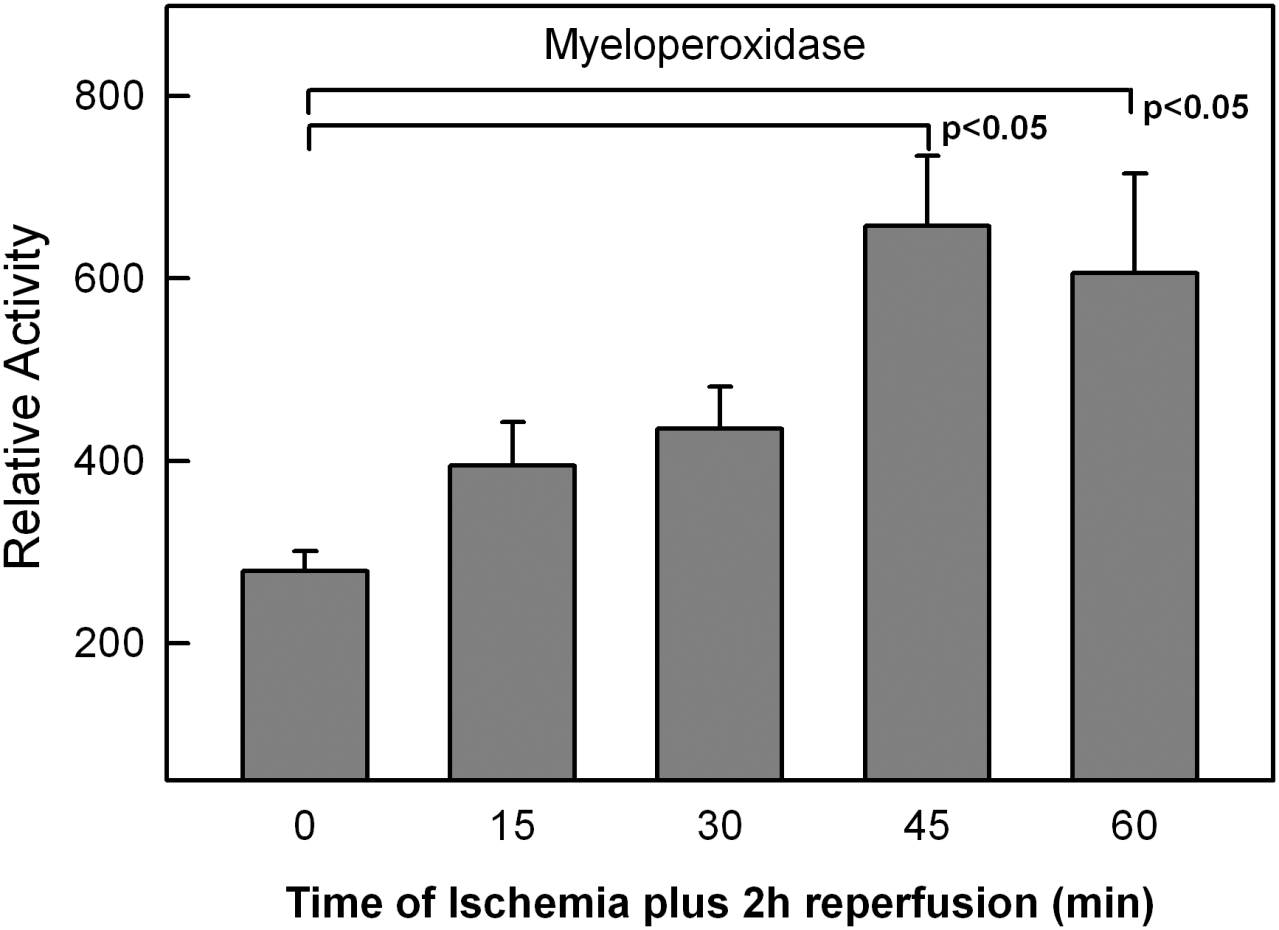
Small bowel myeloperoxidase (MPO) levels following acute intestinal ischemia/reperfusion (AII/R) injury. Ischemia times were set up to represent a time course of intestinal ischemia from 0–60 min all followed by 2 hrs of reperfusion. Myeloperoxidase activity, used a surrogate marker of tissue injury, demonstrates increased intestinal injury with increasing ischemia time. MPO activity is expressed as the maximum rate of OD_460_ change (V_max_) normalized to the protein concentration ([protein]) of each tissue sample. Data for each animal group is expressed as the mean MPO activity with the standard error of the mean. Statistical significance was determined at p<0.05 via one-way ANOVA (nonparametric) test.

A time course for intestinal ischemia was performed at 0, 15, 30, 45, 60 minutes followed by 2 hours of reperfusion. There was an increase in MPO levels with increasing ischemia times (Fig 2). Significance in MPO activity was reached at 45 min and 60 min of ischemia compared to shame. Three time points were subsequently chosen to perform NMR analysis, sham (no ischemia), early (30 min) and late (60 min) of acute intestinal ischemia followed by 2 hours of reperfusion.

### Metabolites identified in urine

**Fig 3:**
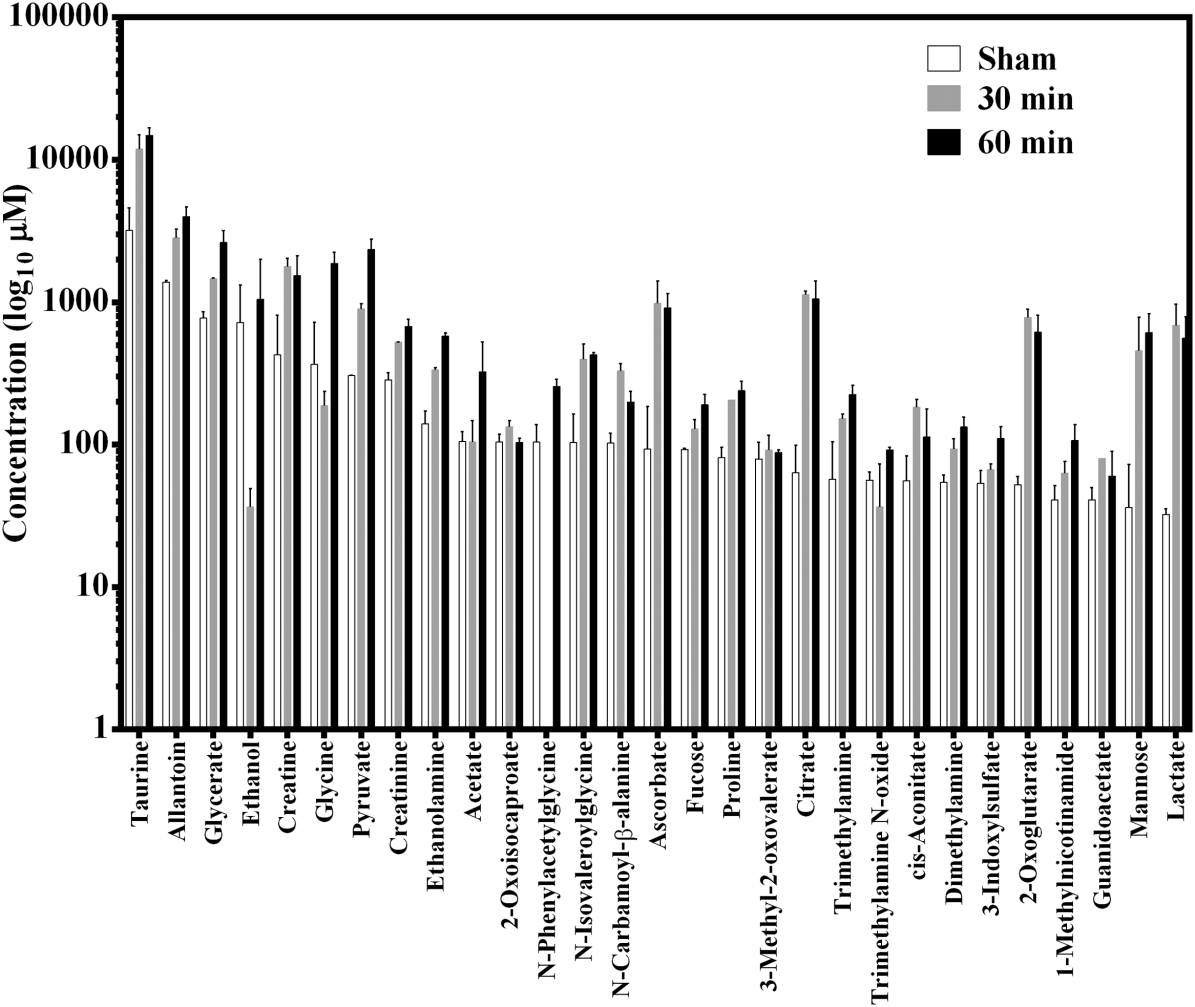
Metabolite concentrations from sham and acute intestinal ischemia/reperfusion (AII/R). Urine from animals in sham, 30 min and 60 min ischemia (all followed by 2 hrs of reperfusion) were analyzed by NMR spectroscopy. The raw concentrations of the top 29 metabolites are illustrated. These were selected to illustrate the concentration of lactate, a common blood marker for tissue injury. Data is expressed as mean ± standard error. None of the metabolite concentrations were found to be statistically significant from the sham-treated animals.

**Fig 4:**
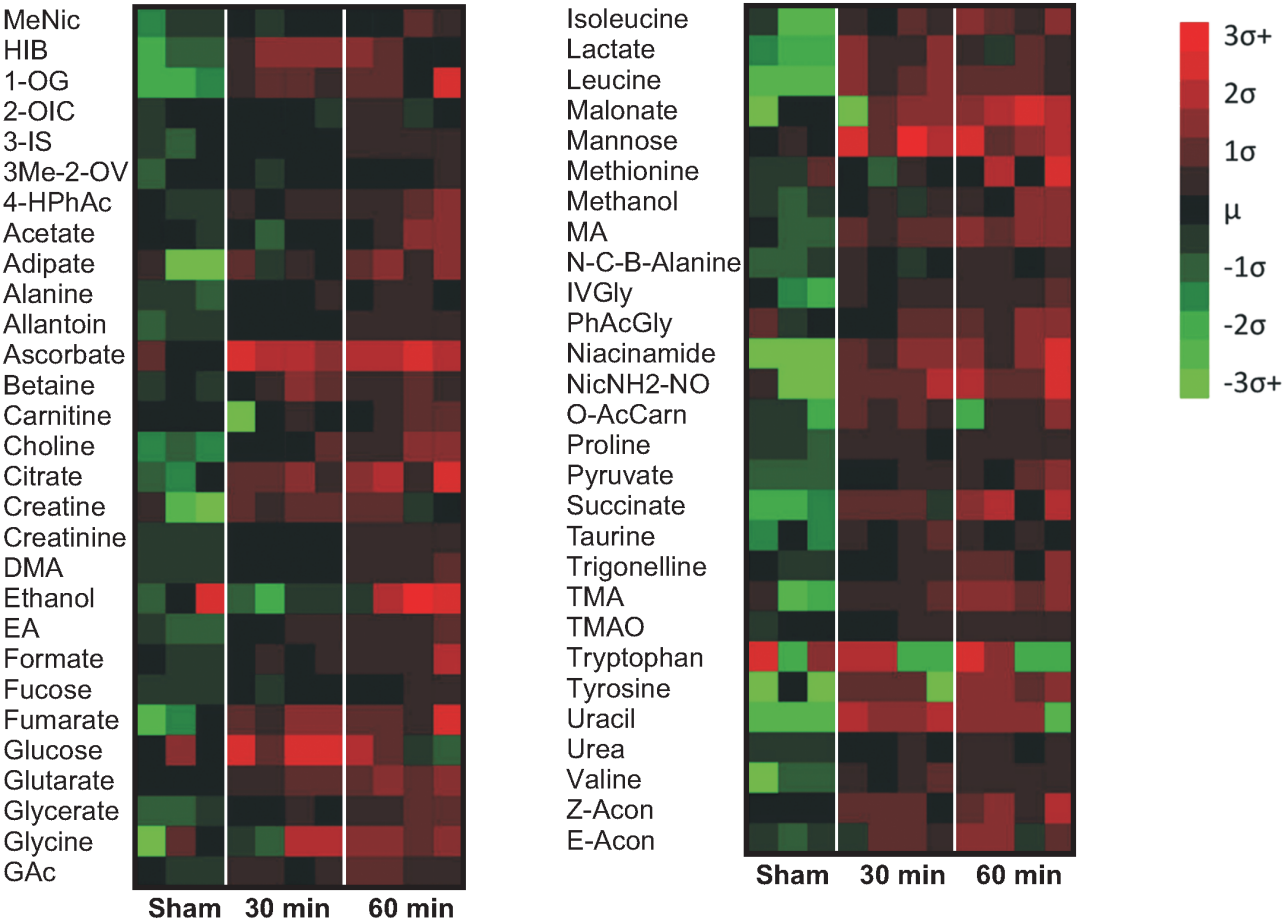
Heat map profile of metabolites from sham and acute intestinal ischemia/reperfusion (AII/R) animals. There were 57 compounds that were identified in the urine of mice in the sham and AII/R-treated groups. These metabolites belong to a variety of biochemical sub-groups. Metabolite concentrations across all animals were averaged and the resulting difference (± standard deviation; σ) displayed as a color index of red (>mean) or green (<mean). Sham (#1–3), 30 min (#4–7) and 60 min (#8–11) intestinal ischemia animals.

Metabolite concentrations in the urine samples (sham and AII/R injury) were derived from NMR spectra analysis using a library of 297 compounds. Altogether, 57 compounds were identified in the spectra ranging from alcohols, amino acids, fatty acids, and lipids. The top 29 metabolites at baseline were illustrated from highest to lowest with lactate being the lowest (Fig 3). The concentrations of most of the metabolites were higher in the 30 min and 60 min ischemia groups compared to the sham controls (see S1 Table in the supporting data). To better visualise changes in metabolites concentration, we illustrated the results using a heat map (Fig 4). The heat map was created with the standard scores for the 57 metabolites from sham versus 30 min and 60 min of AII/R injury. The ‘standard score’ shows how the concentration of each metabolite changes relative to the mean value of the entire pooled group. Red color indicates that there was a greater abundance of that specific metabolite compared to the mean of the group; while green is indicative of lower metabolite levels. AII/R had a direct effect on the concentrations of metabolites as there was a clear visual difference between the sham animals (Fig 4, column 1–3) and the 30 min (column 4–7) and 60 min of AII/R (column 8–11). Compared to the AII/R treated mice, the concentrations of metabolites in the sham group were relatively lower than the 30 min and 60 min ischemia groups.

### Principal component analysis (PCA) of varying times of intestinal ischemia

**Fig 5:**
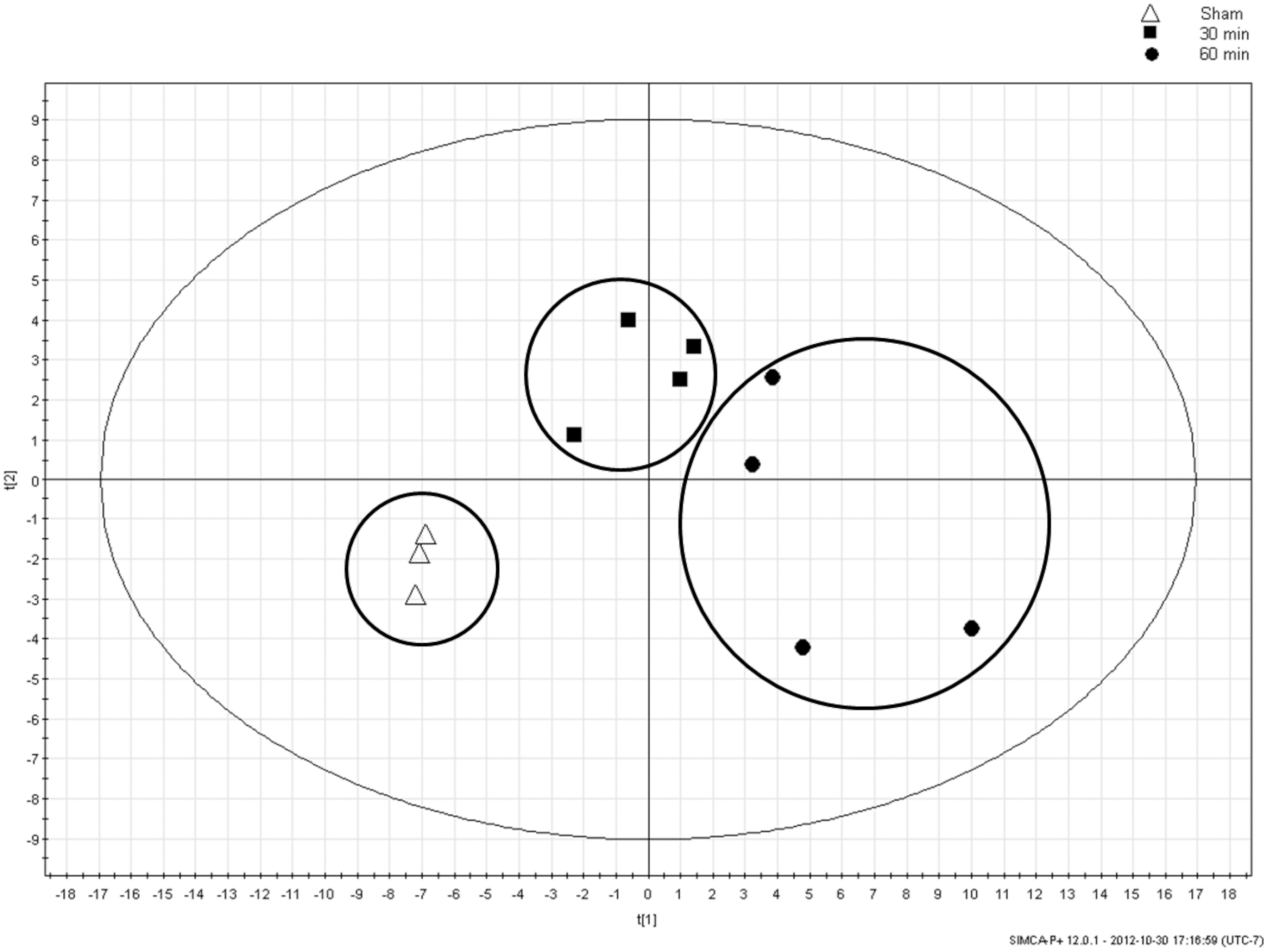
Principal component analysis (PCA) of animal groups with varying times of intestinal ischemia. Metabolites identified in the NMR spectra were condensed by PCA analysis into 2 variables (t[1] (57.3%) and t[2] (12.1%)). Analysis demonstrated a clear separation between Sham (Δ), 30 min ischemia (■) and 60 min ischemia (●) animal groups.

PCA is an unsupervised multivariate statistical analysis method that strives to reduce the dimensionality of multidimensional datasets [32]. PCA was used here to identify the interrelatedness within the groups. In other words, if left unsupervised, do the 3 groups—sham, early and late AII/R—group together based on their metabolite measurements? The PCA plot showed clustering of the AII/R treatment groups which separated from the sham animals (Fig 5). This finding corroborated our heat map data where a visual difference between the AII/R treatment groups and sham was observed.

### Orthogonal projection to latent structures-discriminant analysis (OPLS-DA)

**Fig 6:**
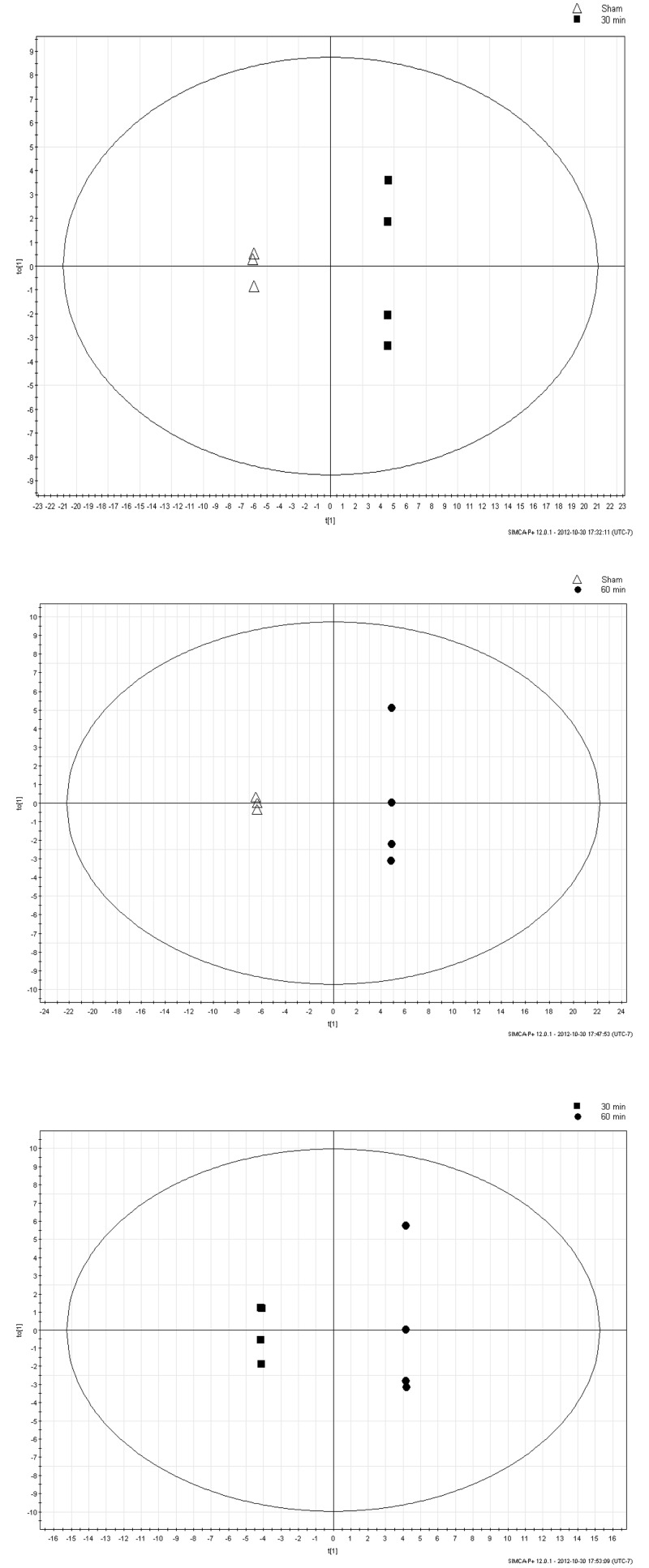
Orthogonal projection to latent structures-discriminant analysis (OPLS-DA). The OPLS-DA of sham versus 30 min of intestinal ischemia (a), sham versus 60 min of ischemia (b), and 30 versus 60 min of ischemia (c) show a clear separation of the metabolites with sham, early and late ischemia times.

OPLS-DA is a regression extension of PCA. OPLS-DA is a supervised analysis which uses class information in an attempt to maximize the separation between the groups of observations. OPLS-DA of all data derived from measured urine metabolite concentrations of sham and AII/R animals is presented in Fig 6. The groups of sham, 30, and 60 min were assigned to the program to mathematically determine the largest differences in the metabolites to separate these groups. OPLS-DA demonstrated metabolite differences with progression of early (30 min) to late (60 min) intestinal ischemia.

### Variable Importance in the Projection (VIP) plot to identify metabolites

**Fig 7:**
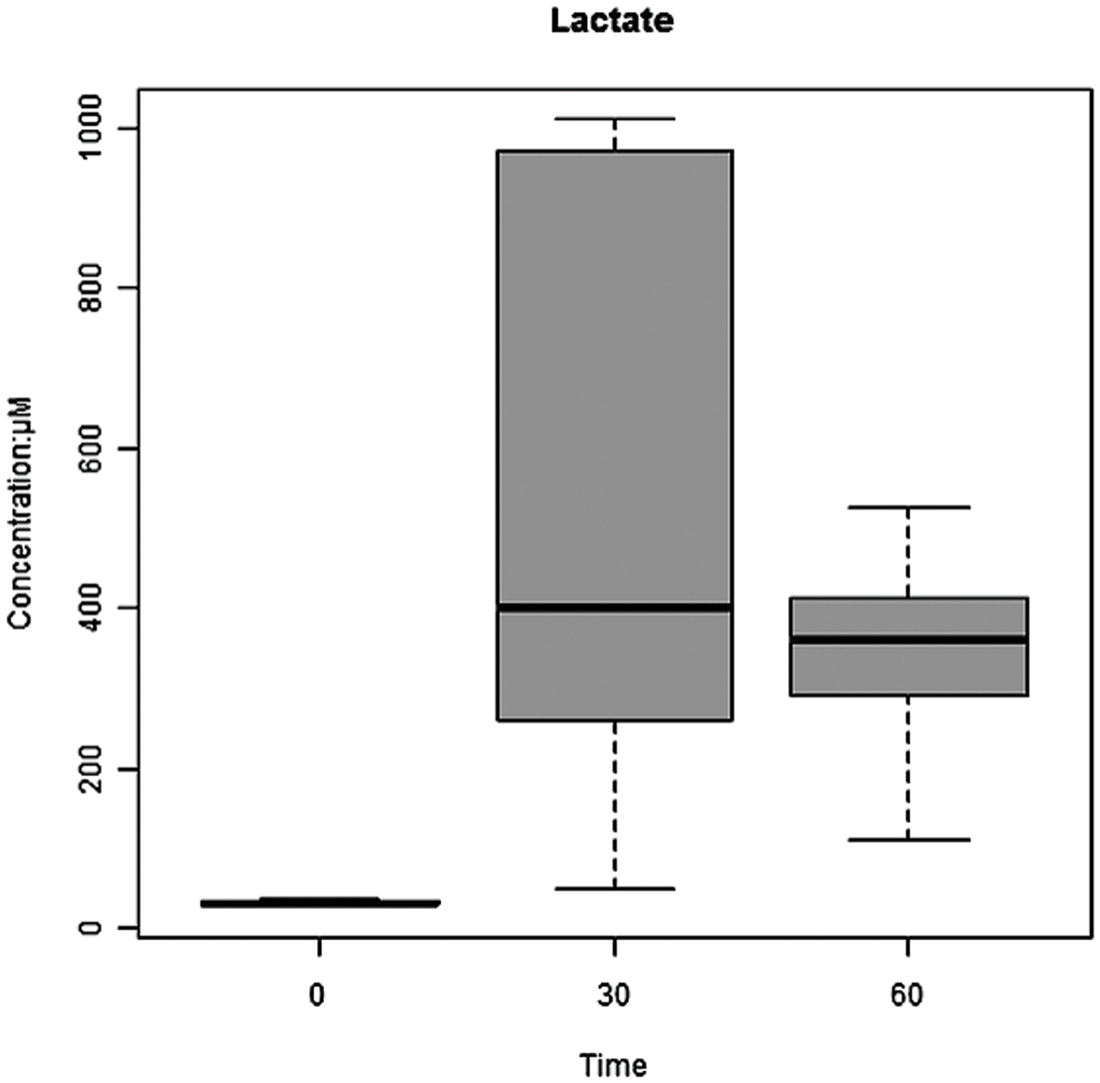
Lactate concentrations cannot differentiate early from late intestinal ischemia/reperfusion (AII/R) injury. Mean lactate levels increased with AII/R injury but did not differentiate early (30 min) from late (60 min) intestinal ischemia.

Following the illustration that the 3 different treatment groups (sham, 30 min and 60 min) separated and clustered together, we wanted to identify which metabolites were more likely to have an impact on predicting ischemic injury. VIP plots are used to show which metabolites have the greatest overall impact on shaping the model of AII/R injury. A Z-score was used to standardize the VIP values in terms of the number of standard deviations the mean VIP number is above a zero value. Any value above the Z-score of 1.64 was associated with having a statistically significant impact on the overall model (see Table 1 for comparisons between sham vs. 30min and sham vs. 60min). Some common metabolites such as allantoin, creatinine, proline, and methylamine were found to have an important effect in differentiating AII/R injury from sham. It is important to note that although lactate is used as a serum clinical marker for AII/R injury, urine lactate measurements did not play an important in predicting intestinal ischemia in our model. Analysis of urine lactate showed a trend towards significant difference (*P* = 0.057) between sham and 30 min or 60 min of AII/R (Fig 7).

**Table 1:** Analysis of Variable Importance in Projection (VIP) metabolites at 30 and 60 min.

Metabolites		Sham vs 30m		Sham vs 60 m	
		Z-score	P value	Z-score	P value
**Amines**	**Methylamine**	3.49	0.001	3.54	0.001
	**Ethanolamine**	2.37	0.01	2.40	0.01
	**Dimethylamine**	2.41	0.01	4.17	0.001
	**Trimethylamine**	0.92	0.18	1.91	0.05
**Amino Acids**	**Valine**	1.67	0.05	3.48	0.001
	**Leucine**	2.34	0.01	2.02	0.05
	**Isoleucine**	2.26	0.01	2.63	0.01
	**N-Isovaleroylglycine**	1.93	0.05	2.03	0.05
	**Proline**	2.65	0.01	3.14	0.001
	**Alanine**	2.56	0.01	1.77	0.05
	**Tyrosine**	0.76	0.22	2.29	0.01
**Lipid Metabolism**	**Choline**	1.31	0.10	2.65	0.01
	**Glycerate**	2.45	0.01	4.04	0.001
	**Glutarate**	1.66	0.01	2.17	0.05
**Purines**	**Allantoin**	4.61	0.001	6.62	0.00001
	**Urea**	2.77	0.01	2.08	0.05
	**Creatinine**	2.97	0.001	5.25	0.001
	**Creatine**	1.62	0.05	0.52	0.30
**Others**	**4-Hydroxyphenylacetate**	1.78	0.05	2.05	0.05
	**Fucose**	2.57	0.01	2.33	0.01

A Z-score was used to standardize the VIP values. Any value above the Z-score of 1.64 was associated with having a statistically significant impact on the overall model.

## Discussion

Acute intestinal ischemia/reperfusion (AII/R) broadly affects critically ill patients in the areas of trauma, transplantation, cardiac surgery, shock, and sepsis. AII/R injury is associated with high mortality rates ranging from 60–90% which has not changed in the last 70 years [6–8]. The high mortality is attributable, in part, to the lack of a predictive diagnostic test that can facilitate the early detection in those at high-risk for intestinal ischemia. This is particularly relevant in those who are critically ill where the classic symptoms of abdominal pain are obfuscated due to sedation and mechanical ventilation [33]. Earlier diagnosis can result in either earlier treatment potentially preventing the need for surgery or if surgery was indicated, it would occur earlier, reducing the morbidity and mortality. We have demonstrated in patients that conventional markers of intestinal ischemia, serum lactate and white blood cell count (WBC) both lack sensitivity and specificity [34], whereas invasive diagnostic tests, such as computer tomography (CT) or angiography, expose patients to the risk of contrast-induced nephropathy and are also not definitive [35].

The aim of this study was to evaluate the potential for diagnosing AII/R using metabolic profiling. Here we used an unsupervised PCA plot to convert a large pool of metabolites that were condensed into a 2-dimentional data set. We illustrated a clear separation between sham controls and AII/R treated mice at 30 min and 60 min, demonstrating that urinary metabolite concentrations are effective at distinguishing AII/R progression. PCA data derived from measured urinary metabolite concentration has the power to differentiate a unique metabolite fingerprint based on the duration of ischemia. Serum lactate is normally used in clinical medicine to ascertain ischemia, however in our model, urine lactate did not play a significant role as a predictive metabolite in differentiating between sham and intestinal ischemia. Previous studies conducted by us and others have shown that urine lactate is not a good predictor of intestinal ischemia [29, 34, 36]. Only one paper has examined metabolite makers in a mouse intestinal ischemia model, using gas chromatography-mass spectrometry, and they corroborate our findings that lactate was not a predictive marker of intestinal ischemia [28]. However, two important distinguishing features in this paper was that metabolomics analysis was done on serum samples, while their model only had an ischemia phase without reperfusion. Our study utilised urine samples and included the important and clinically relevant reperfusion which results in liberation of oxygen free radicals leading to local and distant organ injury [13, 14, 18, 37].

Metabolomics is increasingly being used to study the pathophysiology of gastrointestinal diseases [38]. Though very limited publications have looked at metabolite changes following acute intestinal inflammation, several studies were able to discriminate patients with chronic intestinal inflammation (those with inflammatory bowel disease) from non-inflamed controls (reviewed by De Preter and Verbeke (38)). According to our VIP plot, there were 16 metabolites that played a statistically significant role in directing changes in both 30 and 60 min time points in the AII/R mice compared to the sham controls. The vast majority of these 16 metabolites developed into several common themes with respect to metabolic processes (Table 1). In comparing sham versus 30min AII/R animals, we found allantoin (ranked #1/16 via Z score), methylamine (2/16), creatinine (3/16), and to a lesser extent, urea as the top contributing metabolites in the early acute ischemia. Allantoin is the predominant product of free radical-induced oxidation product of uric acid. Uric acid acts as an antioxidant and upon the availability of biologically relevant oxidants, it is converted into allantoin [39]. Similar to other studies that showed allantoin as a sensitive marker of oxidative stress [39–41], we also show here that AII/R injury results in high levels of allantoin at both ischemia times. Methylamine is a product of gut bacteria metabolising dietary choline and it is lowered upon alterations in gut microbiota composition [42–44]. It is therefore not surprising that after 60 min of ischemia, the levels of methyalamines drops and level of choline increases in our model. We [18] and others [45] have shown that probiotics and preservation of gut microbiota can protect the intestine against ischemia/reperfusion-induced intestinal injury. Interestingly, creatinine was a consistent driving metabolite at both 30 and 60 min; its precursor, creatine was a driving metabolite at 30 min (but not at 60 min). This may indicate an elevation of creatine might be a marker for early I/R while its breakdown product, creatinine remains elevated even during the late ischemia phase (60 min).

Several key amino acids were fundamental in driving overall metabolite changes at both 30 and 60 min. Alanine is an end product of amino acid catabolism at a multitude of metabolic loci, and it is synthesized and secreted in abundance under even the most favourable conditions [46]. Alanine typically increases from intestinal amino acid catabolism. However, here we show that with an increase in ischemia times (from 30 min to 60 min), synthesize of alanine is disrupted and its effect on the ischemic model decreases. We speculate that with prolonged intestinal ischemia might shut down vital metabolomic and catabolism processes in the intestine [47]. As such, we also observed decrease of leucine, and proline following 60 min ischemia. However, we noted an increase in valine and isoleucine at 60 min ischemia. Valine and isoleucine are part of the branched-chain amino acids (BCAA) that promote normal growth, tissue repairs and help prevent the breakdown of muscle. Increase of these BCAA supports the theory that prolonged ischemia shuts down the body’s metabolomic and catabolism processes. Other compounds such as fucose, ethanolamine, methylamine, dimethylamine and 4-hydroxyphenylacetate exhibit a consistent theme of an adverse event leading to cell and tissue degradation. While others have used false discovery rate as a mathematical model to control for false-positive discoveries [48–50], we utilised class membership or family-wise correction via OPLS-DA to better expose separation between the treatment groups. Our aim was to identify differences in metabolites between the groups, however, further studies are needed to generate cross validated predictive models.

Though metabolomics-based strategies provide a powerful tool in modern clinical research and precision medicine, precautionary steps are needed in order to avoid pitfalls. Inconsistent sample collection, storage and subsequent preparation for NMR can alter the concentrations of measured metabolites [51]. Additionally, poor quality of data obtained and subsequent data analysis can hamper the interpretation of the data. Rarely do single metabolomic changes occur in pathology but instead a pattern of changes are observed resulting in the metabolic fingerprint or metabolic profile. For this reason, multivariate statistics are required to analyze the data using individual covariate metabolites as well as interaction terms when necessary. Care must be taken to prevent chance correlations and/or of overfitting of the data.

## Conclusions

This study illustrates that changes in the urinary metabolite profile detected through NMR analysis is effective at detecting AII/R injury and progression in an animal model. The difference in injury was apparent in the PCA and heat map analysis which demonstrated the utility of metabolomics as a diagnostic tool. The unique metabolic fingerprint for AII/R injury makes it an attractive non-invasive and sensitive diagnostic test for AII/R injury. Furthermore, lactate which is a traditional metabolite marker currently used in clinical medicine was not a sensitive predictor of AII/R injury. This proof-of-concept study supports the further examination of NMR technology in a patient population at risk for intestinal ischemia. The discovery of a fingerprint profile of AII/R will be a major advancement in its diagnosis and treatment in critically ill patients.

## Supporting information

S1 Fig Loadings plot. This illustrates the metabolites that play a role in the changes seen following acute intestinal ischemia/reperfusion injury.
(TIF)

S1 Table Concentration of metabolites. The concentrations of the metabolites found in sham controls, 30 min and 60 min ischemia groups are presented.
(DOCX)
