# Supplementary material for: Metabolomic profiling to characterize acute intestinal ischemia/reperfusion injury
Source: PLoS One. 2017 Jun 29;12(6):e0179326. doi: 10.1371/journal.pone.0179326 (PMC5491008; doi:10.1371/journal.pone.0179326)
Supplement: S1 Table — The concentrations of the metabolites found in sham controls, 30 min and 60 min ischemia groups are presented. (DOCX) [file pone.0179326.s002.docx]

**S1 Table. Identified metabolite concentration.**

| **Metabolite** | **Sham** | **30 min** | **60 min** | **P (Sham vs. 30min)** | **P (Sham vs. 60min)** | **P (30min vs. 60min)** |
| --- | --- | --- | --- | --- | --- | --- |
| 1-Methylnicotinamide | 41±10 | 69±7 | 106±24 | 0.1 | 0.06 | 0.3 |
| 2-Hydroxyisobutyrate | 2±1 | 32±7 | 20±7 | 0.06 | 0.03 | 0.4 |
| 2-Oxoglutarate | 52±7 | 758±120 | 1604±808 | 0.06 | 0.06 | 0.5 |
| 2-Oxoisocaproate | 104±14 | 120±11 | 161±24 | 0.6 | 0.2 | 0.2 |
| 3-Indoxylsulfate | 53±13 | 77±6 | 129±10 | 0.2 | 0.06 | 0.03 |
| 3-Methyl-2-oxovalerate | 79±24 | 88±11 | 105±8 | 0.9 | 0.6 | 0.3 |
| 4-Hydroxyphenylacetate | 25±13 | 97±7 | 171±34 | 0.06 | 0.06 | 0.03 |
| Acetate | 105±19 | 114±19 | 380±140 | 0.9 | 0.06 | 0.06 |
| Adipate | 11±11 | 24±6 | 54±11 | 0.3 | 0.1 | 0.1 |
| Alanine | 14±2 | 33±4 | 38±3 | 0.06 | 0.06 | 0.4 |
| Allantoin | 1378±47 | 3055±236 | 4957±252 | 0.06 | 0.06 | 0.03 |
| Ascorbate | 93±93 | 834±206 | 843±175 | 0.06 | 0.06 | 0.9 |
| Betaine | 29±6 | 89±36 | 74±8 | 0.4 | 0.06 | 0.9 |
| Carnitine | 19±2 | 17±6 | 37±5 | 1 | 0.1 | 0.06 |
| Choline | 7±0.6 | 37±10 | 78±14 | 0.06 | 0.06 | 0.1 |
| Citrate | 63±35 | 1186±216 | 2462±998 | 0.06 | 0.06 | 0.5 |
| Creatine | 427±382 | 1944±165 | 1213±406 | 0.06 | 0.2 | 0.3 |
| Creatinine | 285±34 | 558±23 | 837±55 | 0.06 | 0.06 | 0.03 |
| Dimethylamine | 54±7 | 102±9 | 179±19 | 0.06 | 0.06 | 0.03 |
| Ethanol | 718±602 | 67±19 | 1386±604 | 0.2 | 0.6 | 0.06 |
| Ethanolamine | 140±32 | 444±66 | 682±124 | 0.06 | 0.06 | 0.2 |
| Formate | 10±5 | 35±6 | 88±43 | 0.03 | 0.06 | 0.1 |
| Fucose | 93±1 | 142±11 | 216±26 | 0.06 | 0.06 | 0.03 |
| Fumarate | 7±5 | 45±5 | 58±28 | 0.06 | 0.06 | 0.7 |
| Glucose | 14122±13992 | 89848±20638 | 23101±13026 | 0.1 | 0.4 | 0.06 |
| Glutarate | 26±26 | 149±22 | 202±26 | 0.06 | 0.06 | 0.1 |
| Glycerate | 773±83 | 1765±181 | 3533±352 | 0.06 | 0.06 | 0.03 |
| Glycine | 367±358 | 1208±594 | 1325±109 | 0.2 | 0.06 | 1 |
| Guanidoacetate | 41±9 | 53±21 | 116±9 | 0.3 | 0.06 | 0.06 |
| Isoleucine | 4±4 | 24±3 | 43±6 | 0.06 | 0.06 | 0.09 |
| Lactate | 32±3 | 660±193 | 239±66 | 0.06 | 0.06 | 0.3 |
| Leucine | 0±0 | 41±8 | 28±3 | 0.06 | 0.06 | 0.3 |
| Malonate | 14±7 | 54±19 | 164±30 | 0.3 | 0.06 | 0.03 |
| Mannose | 36±36 | 613±194 | 336±77 | 0.06 | 0.06 | 0.5 |
| Methanol | 73±38 | 36±18 | 647±295 | 0.6 | 0.1 | 0.09 |
| Methionine | 29±6 | 63±13 | 140±41 | 0.03 | 0.06 | 0.2 |
| Methylamine | 15±8 | 151±21 | 261±33 | 0.06 | 0.06 | 0.06 |
| N-Carbamoyl-β-alanine | 102±18 | 249±52 | 339±30 | 0.1 | 0.06 | 0.3 |
| N-Isovaleroylglycine | 103±61 | 419±48 | 445±51 | 0.06 | 0.06 | 0.9 |
| N-Phenylacetylglycine | 104±35 | 113±65 | 236±41 | 1 | 0.1 | 0.2 |
| Niacinamide | 0±0 | 60±14 | 86±33 | 0.06 | 0.06 | 0.9 |
| Nicotinamide N-oxide | 12±12 | 85±18 | 119±34 | 0.03 | 0.06 | 0.4 |
| O-Acetylcarnitine | 3±2 | 14±2 | 12±6 | 0.06 | 0.3 | 0.7 |
| Proline | 81±15 | 202±3 | 292±28 | 0.06 | 0.06 | 0.03 |
| Pyruvate | 306±2 | 1408±298 | 2395±665 | 0.06 | 0.06 | 0.3 |
| Succinate | 9±1 | 157±37 | 267±73 | 0.06 | 0.06 | 0.2 |
| Taurine | 3189±1402 | 14322±1924 | 10443±1001 | 0.06 | 0.06 | 0.2 |
| Trigonelline | 14±7 | 46±3 | 88±17 | 0.03 | 0.06 | 0.3 |
| Trimethylamine | 57±48 | 175±23 | 340±27 | 0.2 | 0.06 | 0.03 |
| Trimethylamine N-oxide | 56±8 | 64±22 | 93±6 | 0.4 | 0.06 | 0.3 |
| Tryptophan | 30±19 | 25±14 | 29±19 | 0.9 | 1 | 1 |
| Tyrosine | 7±7 | 47±16 | 92±11 | 0.2 | 0.06 | 0.1 |
| Uracil | 0±0 | 78±6 | 51±17 | 0.06 | 0.1 | 0.1 |
| Urea | 38271±1423 | 84060±6946 | 113780±13752 | 0.06 | 0.06 | 0.3 |
| Valine | 4±2 | 33±7 | 28±2 | 0.06 | 0.06 | 1 |
| cis-Aconitate | 56±28 | 148±51 | 307±106 | 0.3 | 0.06 | 0.3 |
| trans-Aconitate | 28±5 | 89±20 | 152±44 | 0.06 | 0.2 | 0.3 |

Data presented as mean±SD. Groups were compared via Mann-Whitney *U* test.
